# GenomeDelta: detecting recent transposable element invasions without repeat library

**DOI:** 10.1186/s13059-024-03459-5

**Published:** 2024-12-18

**Authors:** Riccardo Pianezza, Anna Haider, Robert Kofler

**Affiliations:** 1 Institut für Populationsgenetik, Vetmeduni Vienna, Veterinärplatz 1, 1210 Vienna, Austria; 2 Vienna Graduate School of Population Genetics, Vetmeduni Vienna, Vienna, Austria

**Keywords:** Transposable elements, Repeat library, Horizontal gene transfer, Lateral gene transfer, Non-model organisms, Genome assemblies, Short reads

## Abstract

We present GenomeDelta, a novel tool for identifying sample-specific sequences, such as recent transposable element (TE) invasions, without requiring a repeat library. GenomeDelta compares high-quality assemblies with short-read data to detect sequences absent from the short reads. It is applicable to both model and non-model organisms and can identify recent TE invasions, spatially heterogeneous sequences, viral insertions, and hotizontal gene transfers. GenomeDelta was validated with simulated and real data and used to discover three recent TE invasions in *Drosophila melanogaster* and a novel TE with geographic variation in *Zymoseptoria tritici*.

**Supplementary Information:**

The online version contains supplementary material available at 10.1186/s13059-024-03459-5.

## Background

Transposable elements (TEs) are short DNA sequences capable of increasing their copy numbers within a host genome. They are common in many organisms and often make up a large part of their genome [1, 2]. While some TEs may confer benefits to hosts [3, 4], the majority of TE insertions are likely neutral or deleterious [5, 6].

Consequently, host genomes have evolved elaborate defense mechanisms, frequently involving small RNAs [7]. TEs can evade host silencing through horizontal transfer (HT), i.e., the transmission to naive species that lack the TE [8–11]. HT is a common phenomenon in prokaryotes [12], but recent studies suggest that HT (especially of TEs) is also prevalent in eukaryotic organisms [8, 13].

The evidence for HT of TEs has typically been indirect. Such evidence includes a patchy distribution of the TE among closely related species or a high similarity between the TE of the donor and recipient species, which is frequently quantified by the synonymous divergence of the TE [14, 15].

However, for TEs that have spread very recently, direct evidence of the recent invasion can be obtained, for example, when the sequence of the TE is absent in older samples but present in more recently collected ones. To illustrate, the *P-element* invaded *D. melanogaster* populations between 1950 and 1980 [16, 17]. Consequently, sequences with similarity to the *P-element* are absent in natural *D. melanogaster* strains collected before 1950 but present in strains collected after 1980. It is possible that such recent invasions are common. In *D. melanogaster*, an important model organism for studying TE dynamics, 11 different TE families invaded natural populations during the last 200 years [9, 16–22]. It is feasible that other organisms might also experience a high rate of recent invasions.

To obtain direct evidence for recent TE invasion, it is necessary to compare the sequencing data from old and recent samples. Recent samples can be collected from natural populations, whereas old samples may be derived from several sources, including old laboratory strains, genomes of historical specimens from museums, or ancient DNA extracted from archeological remains [20, 23, 24]. The number of species with sequencing data from historical specimens is rapidly increasing [25] (for example, *Anopheles* sp. [26], *Apis mellifera* [27], *Columba livia* [28], and *Canis lupus* [29]). Therefore, it is in principle feasible to discover direct evidence for recent TE invasions in an increasing number of species. However, discovering recent invasions with existing approaches typically requires comparing the copy numbers of known TE families in old and young samples (e.g., [9, 20]). These approaches thus require prior knowledge of the sequences of the TEs, i.e., a repeat library. Generating repeat libraries is notoriously difficult, requiring extensive manual curation [30–32]. This issue is further compounded by the fact that even for the few species for which a high-quality repeat library is available, the library may be incomplete and not contain the sequences of TEs that have spread very recently. For example, the high-quality repeat library of *D. melanogaster* [33] lacks the sequence of the retrotransposon *Spoink*, which spread in natural populations between 1983 and 1993. This is because the reference strain used for generating the repeat library was likely collected prior to that period. This is part of the reasons why the *Spoink* invasion was only recently discovered, several years after the invasion [21].

The development of an approach that enables identification of recent TE invasions independent of repeat libraries would represent a substantial conceptual advance in the field. For this reason, we developed GenomeDelta. GenomeDelta is based on the idea that recent invasions will lead to sequences that are present in recently collected samples (i.e., after the invasion) but absent in old samples (i.e., before the invasion). As input, GenomeDelta requires a high-quality assembly (ideally a long-read assembly) of the recently collected sample and short-read data of the old sample. GenomeDelta then identifies sequences that are present in the assembly but absent in the short-read data. As this approach does not require prior knowledge about the sequences, it allows to comprehensively identify sample-specific sequences (e.g., TEs that invaded recently) in model and non-model organism. Importantly, GenomeDelta is not designed to detect copy number differences among samples or differences in the insertion sites. Apart from finding recent TE invasions, GenomeDelta may also be used to detect sequences showing a geographically heterogeneous distribution, such as the TE *Styx* in *Z. tritici* [34], recent endogenous virus insertions [35], and recent lateral gene transfer [36]. We thoroughly validated our novel tool with simulated and real data. We also provide a detailed manual and a walkthrough. Finally, we show that GenomeDelta can be used to gain novel biological insights. With GenomeDelta, we discovered three novel TE invasion in *D. melanogaster* in the last three decades and a novel TE (*Rosetta*) with a spatially heterogeneous distribution in *Z. tritici*.

## Results

### GenomeDelta

We developed GenomeDelta (GD) to identify genomic sequences that are present in one sample (*P* presence) and absent in another sample (*A* absence). Given the two sets of genomic sequences "*P*" and "*A*", GenomeDelta aims to identify the set of sample-specific sequences $P-A$. Note that GenomeDelta does not compute the set $A-P$.

**Fig. 1:**
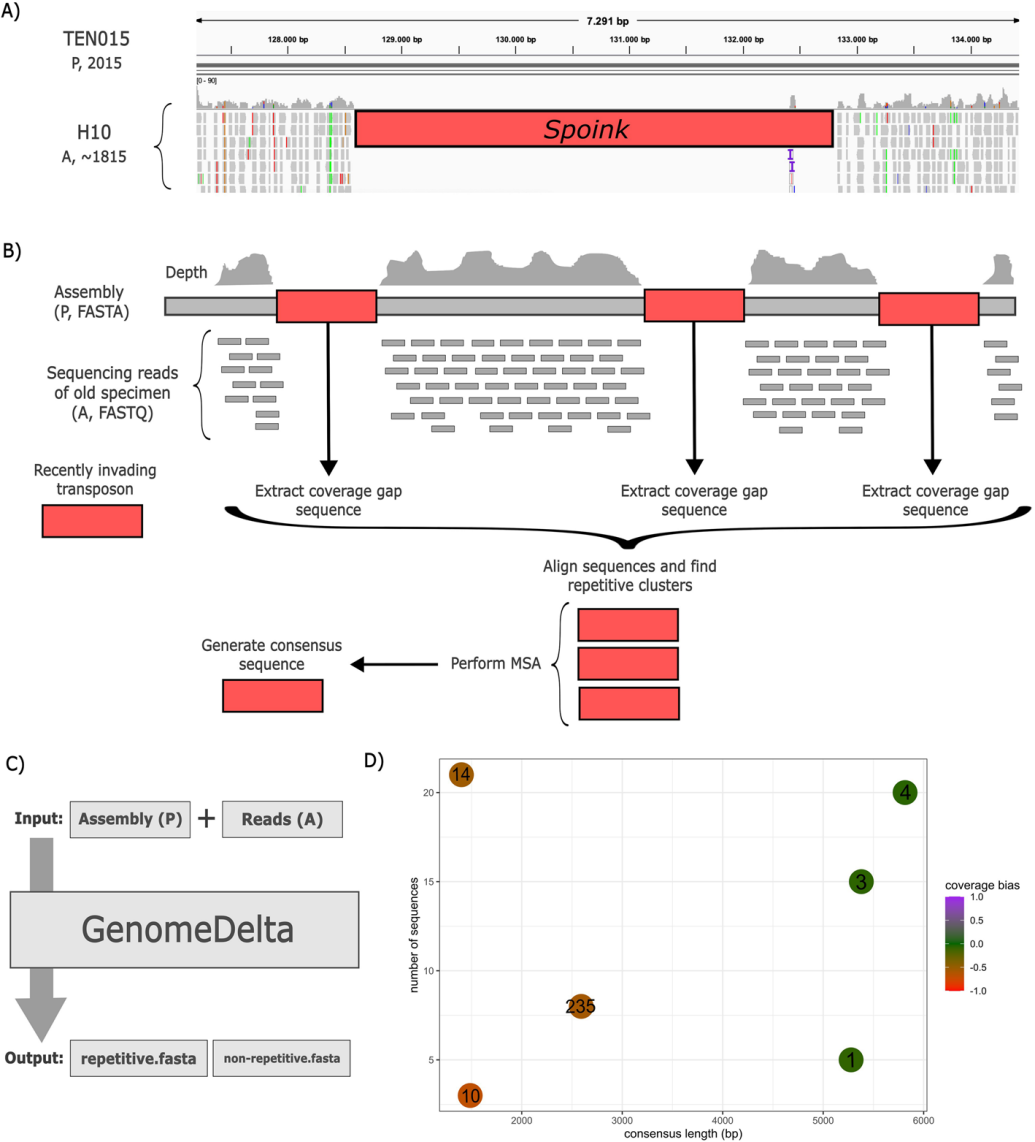
Overview of GenomeDelta. **A** Recent TE invasions will lead to coverage gaps when reads of a sample collected before the invasion (H10, collected in 1815) are aligned to the assembly of a sample collected after the invasion (TEN015, collected in 2015). The coverage gap in this example is due to the retrotransposon *Spoink*, which invaded *D. melanogaster* populations between 1983 and 1993 [21]. **B** Workflow of GenomeDelta. Reads (FASTQ) of a sample *A* (absence) are aligned to an assembly (FASTA) of another sample *P* (presence). The sequences of coverage gaps are extracted, similar sequences are clustered, a multiple sequence alignment is performed, and consensus sequences are generated. Finally, the sequences that are present in *P* but absent in *A* are reported. **C** Overview of the input and output of GenomeDelta. As output, two fasta-files are generated, one with the consensus sequences of repetitive elements and one with the sequences of non-repetitive elements. **D** Graphical output generated by GenomeDelta, providing an intuitive overview of the sample-specific repetitive sequences. The length, the copy number, and the coverage bias (see text) are shown for each identified repetitive sequence

As input, GenomeDelta requires a high-quality assembly for sample *P* and short-read data for sample *A*. GenomeDelta is based on the idea that sequences that are present in *P* and absent in *A* (i.e., $P-A$) can be identified as coverage gaps when short reads of *A* are aligned to an assembly of *P* (Fig. 1A).

One major field of application for GenomeDelta is the identification of novel TE invasions. TE invasions add novel sequences to the genome that are present in samples collected after the invasion (*P*) but absent in samples collected before the invasion (*A*). Another major use-case of GenomeDelta is the identification of sequences (TEs) that are present in one geographic region (*P*) but absent in another (*A*). For example, *KoRV* (koala retrovirus) insertions are present in the genomes of koalas sampled from the North but not in all the koalas from the South of Australia [37]. In summary, GenomeDelta may be used to identify sequences (repetitive or non-repetitive) showing a spatial or temporal heterogeneous presence/absence pattern.

To identify the sample-specific sequences ($P-A$), GenomeDelta aligns the sequencing reads of *A* to the assembly of *P* and computes the coverage. Next, GenomeDelta identifies coverage gaps, extracts the sequences of the gaps, groups them by sequence similarity (e.g., the different insertions of a TE family), performs a multiple sequence alignment (MSA), and reports the consensus sequences (Fig. 1B). Separate results are reported for repetitive and non-repetitive sample-specific sequences (Fig. 1C). Finally, GenomeDelta estimates the reliability for each of these sample-specific sequences ($P-A$) by computing a coverage bias score. The coverage bias is estimated as $bias=2*\frac{f}{g+f}-1$ where *f* is the coverage in the regions flanking the coverage gap (10,000 bp in each direction) and *g* the average genomic coverage. The bias ranges from $-1$ to 1, where 0 indicates an unbiased coverage and $-1$ and 1 a highly biased coverage (either highly decreased or increased; Fig. S1). As output, the sample specific-sequences are provided as two fasta-files, one for the consensus sequences of repetitive elements and one for the non-repetitive sequences. Additionally, a bed file with the genomic coordinates and the coverage bias of each coverage gap is reported. To provide an intuitive graphical overview, GenomeDelta also generates a summary plot, showing for each sample-specific repetitive sequence the copy number, the length, and the coverage bias (Fig. 1D).

GenomeDelta can be easily installed with conda ([38]). A detailed manual and a walkthrough with data from *D. melanogaster* are available. GenomeDelta is distributed under the Open Software License v.2.1.

### Validation

We thoroughly validated GenomeDelta with simulated and real data. For validations with simulated data, we used a chromosome sequence of *D. melanogaster* (chromosome arm 2R) as template and inserted 25 copies of a randomly generated sequence with a length of 5000 bp into this template. We thus obtain an artificial sequence with (*P*) and without TEs (i.e., the template, *A*). Next, we generated artificial short reads for the sequence without TEs (*A*). We simulated artificial reads yielding different coverages and coverage distributions (uniform coverage and random position of reads; Table 1). We also used Gargammel [39] to simulate reads mimicking properties of ancient DNA (i.e., fragmentation, cytosine deamination, bacterial contamination [24]). Finally, we plugged the artificial reads into GenomeDelta to identify the sample-specific sequences. To evaluate the performance of GenomeDelta, we computed the true positive rate, the false positive rate, the length of the identified consensus sequence, and the similarity between the observed consensus sequence and the simulated sequence (Table 1). The 25 artificial insertions were detected in all scenarios. The obtained consensus sequence was 100% identical to the simulated sequence, and the length of the consensus sequence was close to the simulated 5000 bp. With a low coverage, especially when properties of ancient DNA were simulated, the false positive rate was elevated and the length of the consensus sequence deviated from the expected one (i.e., 5000; Table 1). However, with a coverage of $\ge 5$, no false positives were found and the observed length was close to the expected one (Table 1). To test the performance of GenomeDelta with short sequences, we performed an additional validation with a sequence of length 1000 bp. With such a short sequence, GenomeDelta identified more false positive sequences when the coverage was low (i.e., 1x, Table S1). Finally, we simulated reads with different lengths and sequencing error rates. The error rate of the reads and the read length had little impact on the performance of GenomeDelta (Tables S2, S3).

Our validations with simulated data suggest that GenomeDelta accurately identifies sample specific-sequences with a length $>1000$ bp. Furthermore, the short-read data (of sample *A*) ought to have a minimum coverage of 5.

**Table 1:** Validation of GenomeDelta with simulated data. We introduced 25 copies of an artificial TE with a size of 5000 bp into a template sequence and tested if the TE sequence was accurately identified with GenomeDelta. As input, we used the template with the TEs (*P*) and artificial reads simulated from the template without TEs (*A*). We simulated different read coverages using either a uniform or a heterogeneous coverage (random position of reads). We also simulated properties of ancient DNA with Gargammel. We evaluated the number of true positive insertions (*TP*), the number of false positive insertions ($FP_{r}$, $FP_{nr}$: repetitive or non-repetitive), the length of the reported consensus sequence [len. (bp)] as well as the sequence similarity between the reported consensus sequence and the simulated insertion [sim (%)]

Method	Coverage	*TP*	$FP_{nr}$	$FP_{r}$	len. (bp)	sim (%)
uniform	1	25	8	0	5000	100
uniform	5	25	0	0	4998	100
uniform	10	25	0	0	4998	100
random	1	25	607	2	5251	100
random	5	25	0	0	4998	100
random	10	25	0	0	4997	100
gargammel	1	25	2410	17	5435	100
gargammel	5	25	0	0	5000	100
gargammel	10	25	0	0	4997	100

**Fig. 2:**
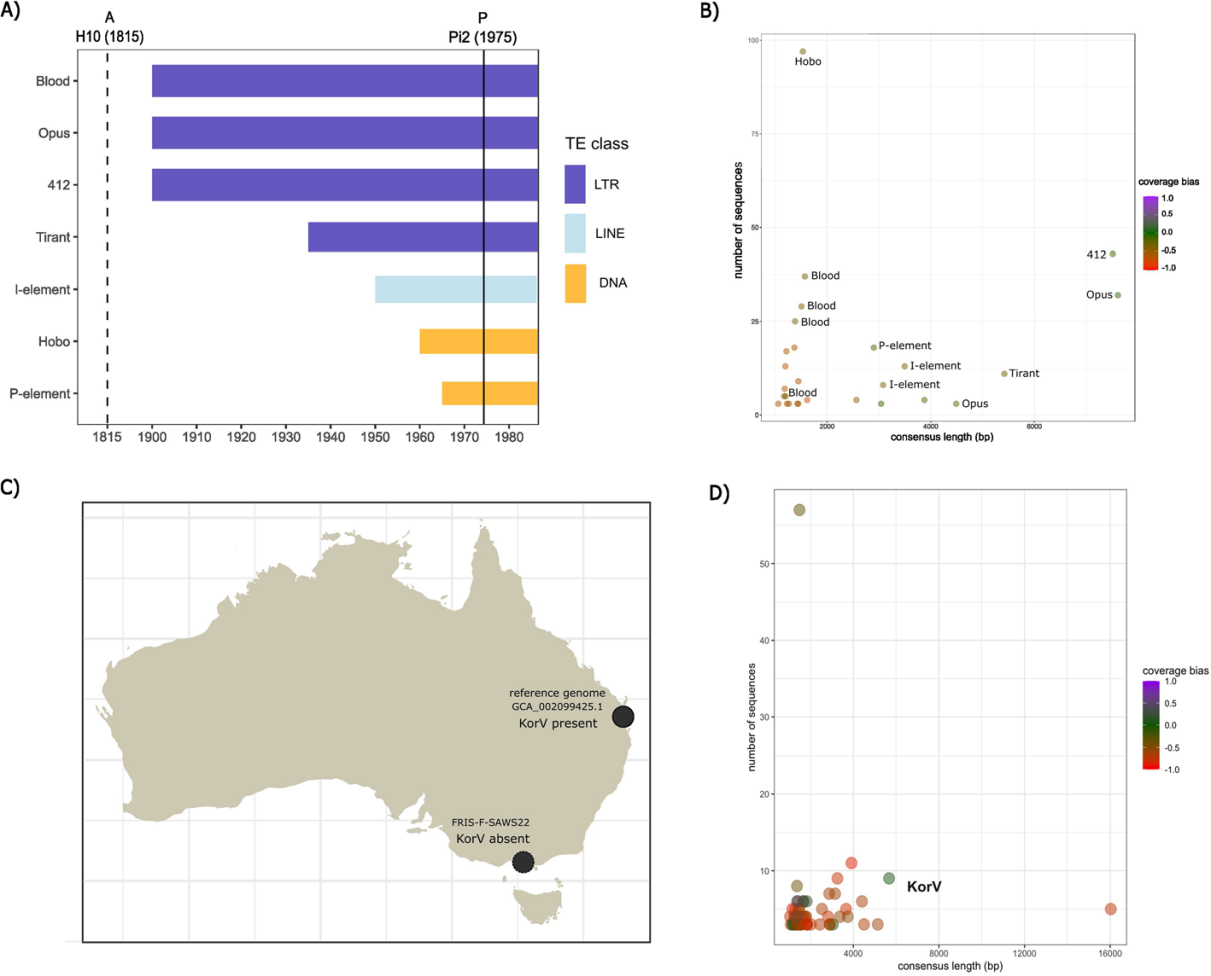
Validation of GenomeDelta with real data. **A** Overview of the invasion history of *D. melanogaster* until 1975 as revealed by previous works. **B** Overview of the sequences identified by GenomeDelta when a sample collected around 1815 (H10) is compared to a strain collected in 1975 (Pi2). Note that the identified sequences correspond to the recent invaders. **C** Previous work shows that KoRV insertions are present in the genomes of koalas sampled in the North but not in the South of Australia. **D** Overview of sequences identified by GenomeDelta when short reads from a southern koala (FRIS-F-SAWS22) are aligned to an assembly of a northern koala (GCA_002099425.1). Note that the best candidate sequence (i.e., coverage bias close to zero) corresponds to KoRV

Next, we validated GenomeDelta with real genomic data from an insect (the fruit fly, *D. melanogaster*) and a mammal (the koala, *Phascolarctos cinereus*).

Based on various approaches, ranging from phenotyping (hybrid dysgenesis) to whole genome sequencing, previous works showed that seven TEs spread in *D. melanogaster* populations between 1800 and 1980 [9, 16–20, 40]. These works showed that *Blood*, *412*, *Opus* spread between 1850 and 1930, *Tirant* around 1935 followed by the *I*-element, *Hobo*, and the *P*-element (Fig. 2A). We tested whether GenomeDelta identifies these known invasions when short-read data of a strain collected around 1815 (H10) are aligned to the assembly of a strain collected around 1975 (Pi2) [41, 42]. Note that the short-read data are derived from a strain kept for $\approx$200 years in a museum, thus the sequencing reads are fragmented with an average size of around 50 bp [41]. GenomeDelta detected 27 repetitive sequences that are specific to Pi2 (Fig. 2B). A blast search of the consensus sequences against a TE library [33, 43] revealed that the 7 TE that invaded *D. melanogaster* recently are the most notable outliers in terms of length, copy numbers, and coverage bias (Fig. 2B). Note that several fragments are reported for some TEs (*Blood*, *I*-element, *Tirant*; Fig. 2B). This can be explained by the fact that degraded fragments of these TEs, likely the remnants of ancient invasions, are present in all genomes, including the genome of the strain sampled at 1815 [9, 20]. Reads derived from these fragments may be mapped on the new TE insertions, thus fragmenting the coverage gap generated by the new insertion. The other sample-specific sequences were less promising, as, for example, seen by the high coverage bias. Closer inspection revealed that many of these sequences were located in telomeric regions where few reads aligned in Pi2 (Table S4). GenomeDelta thus identified the 7 TEs that invaded *D. melanogaster* between 1815 and 1975 based on sequencing reads from historical specimens [41].

We next evaluated the influence of the coverage on the performance of GenomeDelta with real data. The sample used to identify the 7 invasions, H10, yielded a coverage of 33x [41]. We subsampled the number of reads of H10 to coverages of 10x, 5x, and 1x, and again used GenomeDelta to identify sequences specific to Pi2 (Fig. S2). With a coverage of 10x, GenomeDelta identified 5 out of the 7 TEs (*Blood* and *Tirant* were missing). With a coverage of 5x, we identified the same 5 out of the 7 TE, but additionally *Hobo* and *Opus* were fragmented into two clusters. With a coverage of 1x, just one short fragment of the *I-element* was found (Fig. S2). In agreement with the validation with artificial data, the subsampling of real data (from historical specimens) suggest that a coverage $>5$ should be used with GenomeDelta.

We next tested the performance of GenomeDelta with a mammal, i.e., the koala (*Phascolarctos cinereus*). Insertions of the koala retrovirus (KoRV) have been found in the genomes of koalas sampled from the North of Australia but not in koalas sampled from the South [37]. KoRV may be at the transition stage between an exogenous element (e.g., virus) and a vertically transmitted endogenous element (i.e., a transposable element [37]). We tested whether GenomeDelta manages to identify KoRV, by comparing short-read data from a southern koala (FRIS-F-SAWS22 [44]) to the assembly of a northern koala (i.e., the reference genome GCA_002099425.1, which is based on a koala from the Sunshine coast; Fig. 2C). GenomeDelta identified several sample-specific repetitive sequences (Fig. 2D). The most promising sequence (low coverage bias, high copy number, and substantial length) corresponds to KoRV (Fig. 2D). Compared to *D. melanogaster*, we find more false positive sequences (57 in koalas and 11 in *D. melanogaster*; Fig. 2B, D). This can likely be explained by the much larger genome size of koalas as compared to *D. melanogaster* (3500 Mb in koalas and 200 Mb in *D. melanogaster* [45, 46]). As a control, we also compared two koalas from a northern population with GenomeDelta (Sunshine-Coast-M-79817 and the reference genome) and did not find any sequences matching with KoRV (Fig. S3). In agreement with previous work, GenomeDelta thus identified the integration of KoRV in the genomes of northern koalas but not in southern koalas [37].

In summary, we thoroughly validated GenomeDelta with artificial and real data. We also showed that GenomeDelta may be used with historical DNA and that a minimum coverage of 5 should be targeted.

### Novel biological insights

**Fig. 3:**
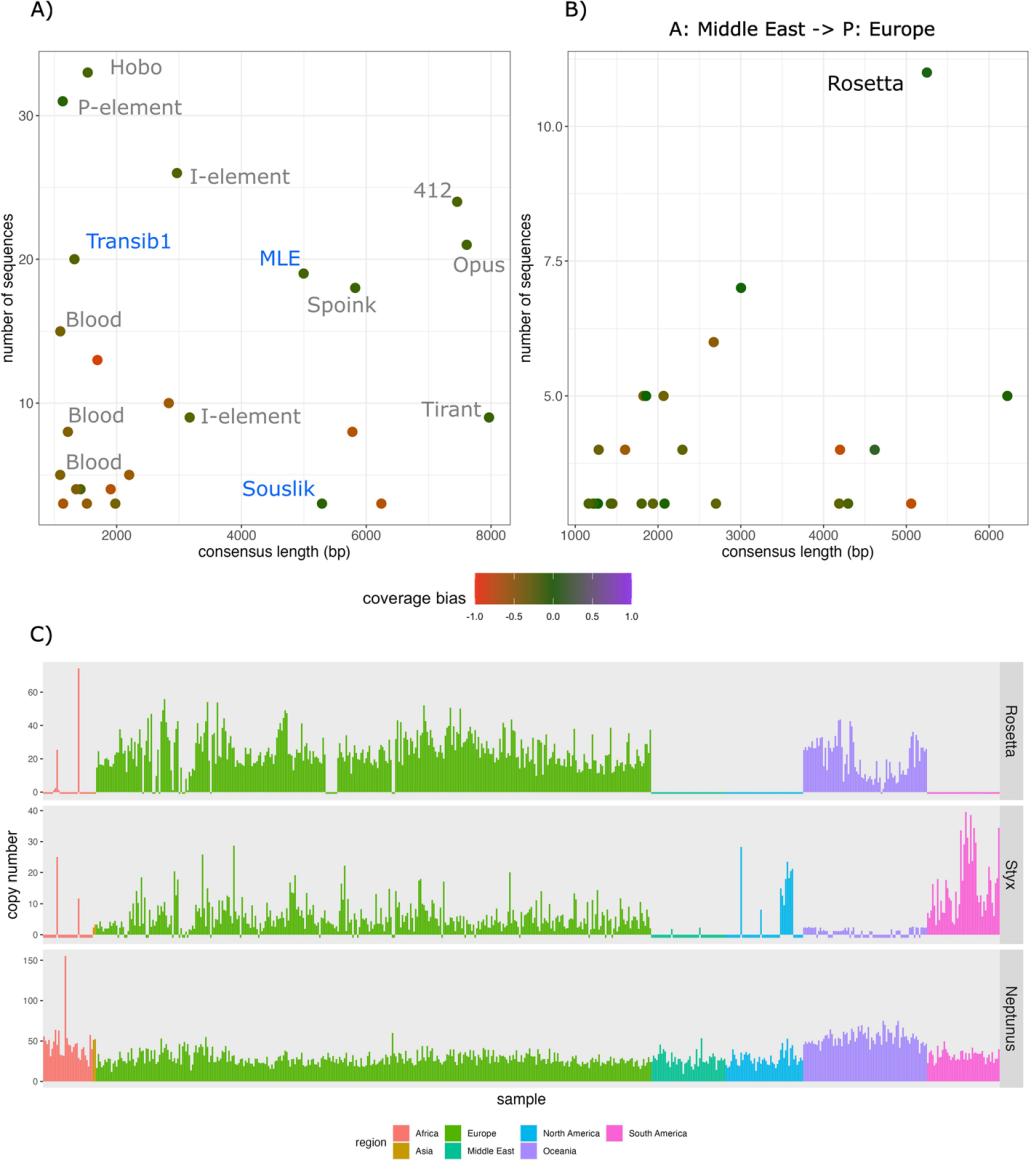
GenomeDelta can be used to generate novel biological insights. **A** GenomeDelta identifies three novel TE invasions in *D. melanogaster* (blue). Short-read data of a strain collected in the early 1800s (*A*: H10) were aligned to the assembly of a strain collected in 2016 (*P*: TOM007). Previously identified TE invasions are in black. **B** GenomeDelta identifies a novel TE (*Rosetta*) with a spatially heterogeneous distribution in *Z. tritici*. Short-read data of a sample from Iran (*A*: SRR5194593) were aligned to the assembly of a sample from the Netherlands (*P*: GCA000219625.1). **C** Copy number of *Rosetta* in *Z. tritici* samples collected from different geographic regions. As controls, a TE with a known spatially heterogeneous distribution (*Styx*) and a TE present in all strains (*Neptunus*) are shown. TE copy numbers were estimated with DeviaTE

Finally, we provide two examples illustrating how GenomeDelta can be used to generate novel biological insights. First, we identified three novel TE invasions in *D. melanogaster* with GenomeDelta (Fig. 2A; [22]). We used GenomeDelta to align reads from a sample collected in the early 1800s (H10) to the assembly of a strain collected in 2016 (TOM007). As expected, we found all the previously described TE invasions that occurred between 1810 and 1975 (Fig. 2A, B) and *Spoink*, which spread between 1983 and 1993 [21]. Surprisingly, GenomeDelta also discovered three novel TE invasions in *D. melanogaster*: *Micropia-like element* (*MLE*), *Souslik*, and *Transib1* (Fig. 3A). The coverage gaps caused by each of these TE families are shown in Fig. S6. These TEs are also present, at least partially, in a repeat library generated from the long-read assemblies of recently collected *D. melanogaster* strains ([47]; for a cross-reference of the TE names between this study and the study of Rech et al. see Table S5). We are describing these novel invasions in detail in a separate work [22]. Here, we use these three novel invasions to showcase how candidates identified with GenomeDelta may be further validated and investigated. We will thus start with the raw results provided by GenomeDelta. As a first step, we investigated the coverage bias. Since the three novel sequences have a low bias (i.e., close to zero), they may be considered promising candidates (Fig. 3A blue). Next, a blast search revealed that these sequences correspond to three different annotated TEs, a “Micropia-like” element (*MLE*) described in *D. melanogaster* (GenBank: MN418888.1), *Transib1* described in *D. melanogaster* and *D. simulans*, and to *Souslik* identified in *D. simulans* (GenBank: BK008880.1). For some TEs, such as *Blood*, the consensus sequence identified by GenomeDelta may be fragmented or incomplete (Fig. 3). To obtain the complete consensus sequence of the novel TEs, we extracted the sequence of each insertion together with the flanking sequences (3000 bp) using bedtools. Next, we performed a multiple sequence alignment of these sequences (TE + flanking region) with MUSCLE and constructed a novel consensus sequence using the GenomeDelta script “MSA2consensus.py,” which employs a simple majority rule for generating consensus sequences. While the consensus sequences of *MLE* and *Souslik* remained largely unchanged, the length of the consensus sequence of *Transib1* increased from 1323 to 3030 bp. This shows that the initial consensus sequence of *Transib1* extracted by GenomeDelta was incomplete. This finding can be attributed to the presence of degraded fragments of *Transib1*, likely remnants of past invasions, in all *D. melanogaster* strains [22, 48]. Reads from ancient *Transib1* fragments may be misaligned to the recent *Transib1* insertions, thus interfering with GenomeDelta’s identification of coverage gaps. Next, we identified the LTR sequence of *MLE* and *Souslik* and the inverted repeat sequences of *Transib1* using BLAST. Based on long-read assemblies, we confirmed the presence of these three TEs in strains collected around 2015 and their absence in strains collected before 1975 [22]. We inferred the exact timing of these three invasions using 585 *D. melanogaster* samples collected during the last 200 years, revealing that *MLE*, *Souslik* and *Transib1* invaded *D. melanogaster* around 1985, 2005, and 2013, respectively [22]. Finally, we aimed to identify the species that acted as donor of the horizontal transfer triggering the invasions, by analyzing the genomes of 1400 arthropod species [22]. The most likely donor species for *MLE* is a *Drosophila* species of the *willistoni* group, while *Souslik* and *Transib1* were likely donated from the sister species *D. simulans* [22]. By identifying three recent TE invasions in *D. melanogaster*, we demonstrated that GenomeDelta can be used to generate novel insights about sequences showing a temporally heterogeneous distribution [22].

We next tested whether GenomeDelta can also be used to generate novel insights about sequences showing a spatially heterogeneous distribution. We utilized the publicly available genomic resources for the important crop pathogen *Zymnoseptoria tritici* (see Additional files 1 and 2), which causes septoria leaf blotch, one of the most important diseases of wheat [49]. *Z. tritici* is native to the Middle East, but spread to all continents between 500 and 200 years ago [50]. Elegant recent work revealed that several TEs (*Styx*, *Juno*, *Deimos*, and *Fatima*) show a spatially heterogeneous distribution in *Z. tritici*, where, for example, the TE *Styx* is present in populations from Europe but not in the Middle East [50] (see also Fig. 3C). The authors attributed this heterogeneous distribution of the TEs to spatial differences in the efficiency of the genomic host defenses against TEs (repeat-induced point mutations [51]). Using GenomeDelta, we aligned short reads from a Middle East sample (SRR5194593) to the assemblies of 15 *Z. tritici* strains collected from diverse continents. As expected, a comparison between the sample from the Middle East and Australia identified TEs that were previously shown to have a geographic heterogeneous distribution (*Styx*, *Juno*, *Deimos*, and *Fatima*, Fig. S4; [50]). Interestingly, by comparing a sample from the Middle East to a sample from the Netherlands, GenomeDelta revealed a novel TE with a heterogeneous distribution, i.e., *Rosetta* (Fig. 3B). Although *Rosetta* has been previously annotated in *Z. tritici*, a spatially heterogeneous composition has not been described before [50, 52]. To further investigate the abundance of *Rosetta* in samples from different geographic regions, we used DeviaTE [53]. DeviaTE estimates the copy numbers of TEs by normalizing the coverage of TEs to the coverage of single copy genes. We found that *Rosetta* is most prevalent in Oceania and Europe but largely absent in Africa, the Middle East, North and South America (Fig. 3C). As control, we also analyzed the abundance of *Styx* with DeviaTE. In agreement with previous work, *Styx* was found in Europe and the Americas but not in Africa and Oceania (Fig. 3C). As a further control, we included a TE (*Neptunus*) present in all populations. A high number of *Neptunus* insertions can be found in all analyzed samples (Fig. 3C). GenomeDelta thus identified *Rosetta* as a novel TE with a spatially heterogeneous distribution in *Z. tritici*. Since the distribution of *Styx* and *Rosetta* varies among regions (e.g., *Rosetta* but not *Styx* is present in Oceania), it is questionable whether differences in the efficiency of the genomic defense as proposed previously [50] can account for the spatially heterogeneous distribution of the TEs. Differences in the host defense ought to affect all TEs equally. An alternative hypothesis might be that *Styx* and *Rosetta* recently spread in *Z. tritici* following a horizontal transfer. The differences in distribution of *Styx* and *Rosetta* can then simply be explained by different geographic origins of the horizontal transfer that triggered the invasions or by differences in invasion routes caused by stochastic migration events transmitting the TE between the populations.

In summary, we showed that GenomeDelta can be used to gain novel biological insight into spatially (*Z. tritici*) and temporally (*D. melanogaster*) heterogeneous distributions of TEs.

## Discussion

In this work, we presented GenomeDelta, a tool designed to detect genomic sequences that are present in one sample but absent in another one. We thoroughly validated GenomeDelta with both artificial and real data and showed that GenomeDelta can be used to generate novel biological insights by detecting recent TE invasions in *D. melanogaster* and identifying a TE with a geographically heterogeneous distribution in *Z. tritici* (Fig. 3).

As major advantage, GenomeDelta can be used to identify sample-specific sequences without prior knowledge about the sequences. Such sample specific sequences are of biological interest and could be generated due to varying processes, ranging from horizontal transfer, to differences in the host defense against foreign DNA and to locally restricted negative selection against some sequences. One important use-case for finding sample specific sequences is the identification of recent TE invasions, where a TE is present in recent but absent in older samples. Previous approaches for finding such recent TE invasions required a comprehensive repeat library, which enables estimating copy number differences of repeats among samples of interest. Creating such repeat libraries is notoriously difficult and requires substantial manual curation [30–32]. Even more problematic is that a single repeat library for a species may not be sufficient, as some TEs may not be present in the library. For example, several TEs that invaded *D. melanogaster* recently (*Spoink*, *MLE*, and *Souslik*) are not present in the high-quality repeat library of *D. melanogaster* (these TEs spread after the strain used for generating the repeat library was collected [22, 33]). A comprehensive identification of all TE invasions would thus require a "pan repeat library" for a species, i.e., a library comprising the repeat sequences of a large number of strains sampled at different times from diverse geographic regions. Generating up-to-date repeat-libraries is thus a major bottleneck with conventional approaches for finding recent TE invasions. The identification of sample-specific sequences with GenomeDelta, independent of a repeat library, is thus a major conceptual advance that enables identifying all recent TE invasions in model and non-model organisms.

GenomeDelta identifies sample-specific sequences ($P-A$) by aligning short reads (sample *A*) to an assembly (sample *P*). Our validations indicate that historical DNA can be used (sample *A*) and that the short-read data (sample *A*) should have a minimum coverage of 5. This coverage requirement of 5 may be a limitation for some projects where shallow sequencing was performed for several samples. In this case, one workaround may be to simply pool the reads of all samples with shared properties (e.g., to pool all samples from the same geographic region or the same decade).

Another limitation is that GenomeDelta solely identifies sequences with qualitative differences, i.e., being present in some samples and absent in others. It will not identify sequences having quantitative differences in copy numbers among samples.

Perhaps the major limitation of GenomeDelta is the requirement for a high-quality genome assembly (sample *P*). High-quality assemblies are necessary, as TE sequences not present in the assembly cannot be detected (e.g., repeat sequences may be missing in low-quality assemblies). It is therefore not possible to identify sequences specific to samples from which a high-quality assembly cannot be generated, such as historical samples, which typically yield only highly fragmented DNA [23]. However, the need for a high-quality assembly is not unique to GenomeDelta; such assemblies are also indispensable for approaches that rely on a repeat library. In fact, without a high-quality assembly, it is impossible to construct a comprehensive repeat library.

Despite these limitations, we anticipate that GenomeDelta may be used for a wide range of applications. Our primary motivation for designing GenomeDelta was to discover recent TE invasions. For example, we used GenomeDelta to discover the three most recent TE invasions in *D. melanogaster* (Fig. 3A; [22]). As another application, GenomeDelta might be used to identify sequences occurring in populations of some regions but not in others. We demonstrate this by using GenomeDelta to discover a novel TE with a geographically heterogeneous composition in *Z. tritici* (*Rosetta*; Fig. 3B, C). Such a geographically heterogeneous distribution of TEs might result from spatial differences in the efficiency of the genomic host defense against TEs [50]. Whether GenomeDelta is best used to search for recent invasions or geographically heterogeneous TE distribution will depend on the research question, the available data/samples, and the population structure of the investigated species. In species with a prominent population structure, there could be some geographic heterogeneity in the TE composition for an extended period, which could be identified with GenomeDelta. On the other hand, a TE may rapidly spread in all individuals of species with little population structure (e.g., *Transib1* in *D. melanogaster* [22]). A heterogenous TE composition may thus be transient, and it may be better to use GenomeDelta to search for recent invasions. Finally, GenomeDelta could also be used to identify sample-specific non-repetitive sequences such as recent endogenous retrovirus insertions [35] and recent lateral gene transfer [36].

## Conclusion

GenomeDelta identifies genomic sequences that are present in one sample and absent in another, such as recent transposable element (TE) invasions, without a repeat library. This represents a significant advantage, as constructing repeat libraries is challenging and the libraries are frequently incomplete, even for well-studied model organisms like *D. melanogaster*. GenomeDelta operates by identifying coverage gaps when short reads from one sample are aligned to a high-quality assembly of another sample.

The tool has a wide range of applications for studying genomic variation. For instance, it has been used to discover three recent TE invasions identified in *D. melanogaster*. Additionally, GenomeDelta has proven effective in identifying sequences with geographically heterogeneous distributions, exemplified by its identification of a TE with a heterogeneous distribution in *Z. tritici*.

GenomeDelta has been validated using both simulated and real data, including historical specimens, demonstrating its reliability and versatility for genomic studies.

## Methods

### Code structure

To identify sequences that are present in sample *P* and absent in sample *A*, GenomeDelta requires an assembly in FASTA format (*P*) and sequencing reads in FASTQ format (*A*).

GenomeDelta proceeds in several steps relying on widely used tools. First, GenomeDelta aligns the reads from *A* to *P* using bwa mem (v 0.7.17, [54]). Note that it is important to use an algorithm that performs a local alignment of the reads (such as bwa mem), otherwise the boundaries of the coverage gaps may be inferred less accurately. The mapped reads are sorted with samtools (v 1.17, [55]), converted to bam files, and the coverage is computed with samtools depth. Next coverage gaps, i.e., regions with zero coverage (default threshold), are identified. To allow for some spurious mapping of reads, GenomeDelta enables merging adjacent coverage gaps separated by a maximum distance of *d* (Fig. S5). All coverage gaps having a minimum size (customizable; per default 1000 bp) are extracted into a fasta-file with bedtools, and a bias score is computed for each gap. The score is computed as $bias=2*\frac{f}{g+f}-1$ where *f* is the coverage in the 10 kb regions flanking the gap (on both sides) and *g* the average coverage of the genome (Fig. S1). Bedtools is used to compute the coverage in the regions flanking the gaps (v 2.30.0, [56]). The bias score ranges from $-1$ to 1, with 0 indicating an unbiased coverage in the flanking regions (i.e., flanking regions have the same coverage as the genomic average). A sample-specific repetitive sequence will lead to several coverage gaps, where each gap correspond to one insertion of the repetitive sequence (e.g., the dispersed insertions of a TE family). GenomeDelta derives a single consensus sequence for each repetitive sequence. To do this, GenomeDelta, extracts the sequence of each coverage gap, clusters them based on a similarity search with BLAST (v 2.6.0, [57]) and a Python script (blast2clusters.py). We use a minimum of 3 sequences per cluster. For each cluster, a multiple sequence alignment (MSA) is generated with MUSCLE (v 3.8.1551, [58]) and the consensus sequence is derived using a Python script “MSA2consensus.py.” The coverage bias of each cluster is calculated as the median of the individual biases of the clustered sequences.

As main output GenomeDelta provides two fasta files: (1) GD-candidates.fasta contains the consensus sequences of the repetitive clusters and (2) GD-non-repetitive.fasta has the sequences of the non-repetitive coverage gaps. Furthermore, a bed file is generated, which includes the genomic coordinates and the coverage bias of each coverage gap. Additionally, GenomeDelta allows to access all generated intermediate files in the output folder. Finally, GenomeDelta provides a plot summarizing the properties of the repetitive clusters, i.e., the number of sequences in the cluster (e.g., the copy numbers of a TE family), the average length of the sequences, and the average coverage bias. The plot is computed with the R package ggplot2 (v 3.4.4, [59]).

### Simulated data

To simulate artificial data, we used the chromosome arm 2R of *D. melanogaster* from the assembly GCA000001215.4 (strain ISO1 [60]) as reference sequence (sample *A*). We randomly inserted repetitive sequences into this reference sequences with SimulaTE (v 1.31, [61]). Artificial reads were generated with a Python script (create-reads.py). Artificial reads that capture properties of ancient DNA were simulated with Gargammel (v3, [39]) using 10% bacterial contamination (*Wolbachia pipientis*, an endosymbiont of *D. melanogaster*), 8% contamination with modern *D. melanogaster* DNA, and 82% DNA of interest. The similarity between the observed consensus sequence and the simulated one was computed with BLAST 2.6.0 [62].

### Validation with real data

To validate GenomeDelta with real data, we used short reads of the strain H10 (1815, Sweden) as well as an assembly of strain Pi2 [41, 42]. We randomly subsampled the reads with Rasusa v0.8.0 [63] to obtain different coverages.

### *Z. tritici*

We used DeviaTE (v0.3.8) [53] to estimate the copy numbers of different TEs, including Rosetta (the novel TE identified by GenomeDelta), in *Z. tritici* strains collected from diverse geographic regions. Short reads were aligned to the sequences of the TEs and to three single copy genes (*MYCGRDRAFT-39655*, *MYCGRDRAFT-70396*, and *MYCGRDRAFT-99758*) with bwa bwasw (version 0.7.17-r1188) [54]. DeviaTE estimates the copy number of a TE by normalizing the coverage of the TE by the coverage of the single copy genes [53].

## Supplementary information

Additional file 1. List of short-reads datasets of *Z. tritici* used in this work.

Additional file 2. List of assemblies of *Z. tritici* used in this work.

Additional file 3.

Additional file 4.

## Data Availability

GenomeDelta is open source and freely available at https://github.com/rpianezza/GenomeDelta. A detailed manual with installation instructions as well as a walkthrough are available. The source code of GenomeDelta as well its validation are also available in Zenodo [64].

## References

[1] Chénais B, Caruso A, Hiard S, Casse N. The impact of transposable elements on eukaryotic genomes: from genome size increase to genetic adaptation to stressful environments. Gene. 2012;509(1):7–15. 22921893 10.1016/j.gene.2012.07.042

[2] Wicker T, Sabot F, Hua-Van A, Bennetzen JL, Capy P, Chalhoub B, et al. A unified classification system for eukaryotic transposable elements. Nat Rev Genet. 2007;8(12):973–82. 17984973 10.1038/nrg2165

[3] Eickbush DG, Eickbush TH. Vertical transmission of the retrotransposable elements R1 and R2 during the evolution of the Drosophila melanogaster species subgroup. Genetics. 1995;139(2):671–84. 7713424 10.1093/genetics/139.2.671 PMC1206373

[4] Nelson J, Slicko A, Yamashita Y. The retrotransposon R2 maintains Drosophila ribosomal DNA repeats. PNAS. 2023;120(23):e2221613120. 37252996 10.1073/pnas.2221613120 PMC10266012

[5] Elena SF, Ekunwe L, Hajela N, Oden SA, Lenski RE. Distribution of fitness effects caused by random insertion mutations in Escherichia coli. Genetica. 1998;102–103:349–58. 9720287

[6] Pasyukova E, Nuzhdin S, Morozova T, Mackay T. Accumulation of transposable elements in the genome of Drosophila melanogaster is associated with a decrease in fitness. J Hered. 2004;95(4):284–90. 15247307 10.1093/jhered/esh050

[7] Sarkies P, Selkirk ME, Jones JT, Blok V, Boothby T, Goldstein B, et al. Ancient and novel small RNA pathways compensate for the loss of piRNAs in multiple independent nematode lineages. PLoS Biol. 2015;13(2):1–20. 10.1371/journal.pbio.1002061 PMC4323106 25668728

[8] Peccoud J, Loiseau V, Cordaux, Gilbert C. Massive horizontal transfer of transposable elements in insects. Proc Natl Acad Sci U S A. 2017;114(18):4721–26. 10.1073/pnas.1621178114 PMC5422770 28416702

[9] Scarpa A, Pianezza R, Wierzbicki F, Kofler R. Genomes of historical specimens reveal multiple invasions of LTR retrotransposons in Drosophila melanogaster populations during the 19th century. bioRxiv. 2023. 10.1101/2023.06.06.543830. 10.1073/pnas.2313866121 PMC11009621 38564639

[10] Kofler R, Hill T, Nolte V, Betancourt A, Schlötterer C. The recent invasion of natural Drosophila simulans populations by the P-element. PNAS. 2015;112(21):6659–63. 25964349 10.1073/pnas.1500758112 PMC4450375

[11] Signor S, Vedanayagam J, Kim B, Wierzbicki F, Kofler R, Lai E. Rapid evolutionary diversification of the flamenco locus across simulans clade Drosophila species. PLoS Genet. 2023;19:e1010914. 10.1371/journal.pgen.1010914 PMC10495008 37643184

[12] Koonin EV, Makarova KS, Aravind L. Horizontal gene transfer in prokaryotes: quantification and classification. Ann Rev Microbiol. 2001;55(1):709–42. 11544372 10.1146/annurev.micro.55.1.709 PMC4781227

[13] Zhang HH, Peccoud J, Xu MRX, Zhang XG, Gilbert C. Horizontal transfer and evolution of transposable elements in vertebrates. Nat Commun. 2020;11(1):1362. 32170101 10.1038/s41467-020-15149-4 PMC7070016

[14] Peccoud J, Cordaux R, Gilbert C. Analyzing horizontal transfer of transposable elements on a large scale: challenges and prospects. BioEssays. 2018;40(2):1700177. 10.1002/bies.201700177 29283188

[15] Wallau GL, Ortiz MF, Loreto ELS. Horizontal transposon transfer in eukarya: detection, bias, and perspectives. Genome Biol Evol. 2012;4(8):801–11. 10.1093/gbe/evs055 PMC3516303 22798449

[16] Kidwell MG. Evolution of hybrid dysgenesis determinants in Drosophila melanogaster. Proc Natl Acad Sci. 1983;80(6):1655–9. 6300863 10.1073/pnas.80.6.1655 PMC393661

[17] Anxolabéhère D, Kidwell MG, Periquet G. Molecular characteristics of diverse populations are consistent with the hypothesis of a recent invasion of Drosophila melanogaster by mobile P elements. Mol Biol Evol. 1988;5(3):252–69. 2838720 10.1093/oxfordjournals.molbev.a040491

[18] Bucheton A, Vaury C, Chaboissier MC, Abad P, Pélisson A, Simonelig M. I elements and the Drosophila genome. Genetica. 1992;86(1–3):175–90. 1281801 10.1007/BF00133719

[19] Bonnivard E, Bazin C, Denis B, Higuet D. A scenario for the hobo transposable element invasion, deduced from the structure of natural populations of Drosophila melanogaster using tandem TPE repeats. Genet Res. 2000;75(1):13–23. 10740917 10.1017/s001667239900395x

[20] Schwarz F, Wierzbicki F, Senti KA, Kofler R. Tirant stealthily invaded natural Drosophila melanogaster populations during the last century. Mol Biol Evol. 2021;38(4):1482–97. 33247725 10.1093/molbev/msaa308 PMC8042734

[21] Pianezza R, Scarpa A, Narayanan P, Signor S, Kofler R. Spoink, a LTR retrotransposon, invaded D. melanogaster populations in the 1990s. bioRxiv. 2023. 10.1101/2023.10.30.564725. 10.1371/journal.pgen.1011201 PMC10965091 38530818

[22] Pianezza R, Scarpa A, Haider A, Signor S, Kofler R. Unveiling the complete invasion history of D. melanogaster: three horizontal transfers of transposable elements in the last 30 years. bioRxiv. 2024. https://www.biorxiv.org/content/early/2024/04/28/2024.04.25.591091. Accessed 1 Dec 2024.

[23] Raxworthy CJ, Smith BT. Mining museums for historical DNA: advances and challenges in museomics. Trends Ecol Evol. 2021. 10.1016/j.tree.2021.07.009. 34456066 10.1016/j.tree.2021.07.009

[24] Orlando L, Allaby R, Skoglund P, Der Sarkissian C, Stockhammer PW, Ávila Arcos MC, et al. Ancient DNA analysis. Nat Rev Methods Prim. 2021. 10.1038/s43586-020-00011-0.

[25] Benham PM, Bowie RC. Natural history collections as a resource for conservation genomics: understanding the past to preserve the future. J Hered. 2023;114(4):367–84. 36512345 10.1093/jhered/esac066

[26] Korlević P, McAlister E, Mayho M, Makunin A, Flicek P, Lawniczak MK. A minimally morphologically destructive approach for DNA retrieval and whole-genome shotgun sequencing of pinned historic dipteran vector species. Genome Biol Evol. 2021;13(10):evab226. 10.1093/gbe/evab226 PMC8536546 34599327

[27] Parejo M, Wragg D, Henriques D, Charrière JD, Estonba A. Digging into the genomic past of Swiss honey bees by whole-genome sequencing museum specimens. Genome Biol Evol. 2020;12(12):2535–51. 32877519 10.1093/gbe/evaa188 PMC7720081

[28] Hernández-Alonso G, Ramos-Madrigal J, van Grouw H, Ciucani MM, Cavill EL, Sinding MHS, et al. Redefining the evolutionary history of the rock dove, Columba livia, using whole genome sequences. Mol Biol Evol. 2023;40(11):msad243. 10.1093/molbev/msad243 PMC10667084 37950889

[29] Bergström A, Stanton DW, Taron UH, Frantz L, Sinding MHS, Ersmark E, et al. Grey wolf genomic history reveals a dual ancestry of dogs. Nature. 2022;607(7918):313–20. 35768506 10.1038/s41586-022-04824-9 PMC9279150

[30] Hoen DR, Hickey G, Bourque G, Casacuberta J, Cordaux R, Feschotte C, et al. A call for benchmarking transposable element annotation methods. Mob DNA. 2015;6(1). 10.1186/s13100-015-0044-6. 10.1186/s13100-015-0044-6 PMC4524446 26244060

[31] Rodriguez F, Arkhipova IR. In: An overview of best practices for transposable element identification, classification, and annotation in eukaryotic genomes. Springer US; 2022. pp. 1–23. 10.1007/978-1-0716-2883-6_1. 10.1007/978-1-0716-2883-6_1 PMC10149145 36449155

[32] Goubert C, Craig RJ, Bilat AF, Peona V, Vogan AA, Protasio AV. A beginner’s guide to manual curation of transposable elements. Mob DNA. 2022;13(1):7. 35354491 10.1186/s13100-021-00259-7 PMC8969392

[33] Quesneville H, Bergman CM, Andrieu O, Autard D, Nouaud D, Ashburner M, et al. Combined evidence annotation of transposable elements in genome sequences. PLoS Comp Biol. 2005;1(2):166–75. 10.1371/journal.pcbi.0010022 PMC1185648 16110336

[34] Feurtey A, Lorrain C, McDonald MC, Milgate A, Solomon PS, Warren R, et al. A thousand-genome panel retraces the global spread and adaptation of a major fungal crop pathogen. Nat Commun. 2023;14(1):1059. 36828814 10.1038/s41467-023-36674-y PMC9958100

[35] Gilbert C, Belliardo C. The diversity of endogenous viral elements in insects. Curr Opin Insect Sci. 2022;49:48–55. 34839030 10.1016/j.cois.2021.11.007

[36] Dunning LT, Olofsson JK, Parisod C, Choudhury RR, Moreno-Villena JJ, Yang Y, et al. Lateral transfers of large DNA fragments spread functional genes among grasses. Proc Natl Acad Sci. 2019;116(10):4416–25. 30787193 10.1073/pnas.1810031116 PMC6410850

[37] Tarlinton RE, Meers J, Young PR. Retroviral invasion of the koala genome. Nature. 2006. 10.1038/nature04841. 16823453 10.1038/nature04841

[38] Anaconda I. Conda: a cross-platform, language-agnostic binary package manager. 2012. Version 4.10.3. https://github.com/conda/conda. Last accessed 1 Dec 2024.

[39] Renaud G, Hanghøj K, Willerslev E, Orlando L. gargammel: a sequence simulator for ancient DNA. Bioinformatics. 2017;33(4):577–9. 27794556 10.1093/bioinformatics/btw670 PMC5408798

[40] Daniels SB, Chovnick A, Boussyy IA. Distribution of hobo transposable elements in the genus Drosophila. Mol Biol Evol. 1990;7(6):589–606. 2178210 10.1093/oxfordjournals.molbev.a040625

[41] Shpak M, Ghanavi HR, Lange JD, Pool JE, Stensmyr MC. Genomes from historical Drosophila melanogaster specimens illuminate adaptive and demographic changes across more than 200 years of evolution. PLoS Biol. 2023;21(10):e3002333. 37824452 10.1371/journal.pbio.3002333 PMC10569592

[42] Wierzbicki F, Schwarz F, Cannalonga O, Kofler R. Novel quality metrics allow identifying and generating high-quality assemblies of piRNA clusters. Mol Ecol Resour. 2022;22(1):102–21. 34181811 10.1111/1755-0998.13455

[43] Chakraborty M, Chang C, Khost D, J Vedanayagam JA, Liao Y, Montooth K, et al. Evolution of genome structure in the *Drosophila simulans* species complex. Genome Res. 2021;31:380–96. 10.1101/gr.263442.120 PMC7919458 33563718

[44] Hogg CJ, Silver L, McLennan EA, Belov K. Koala genome survey: an open data resource to improve conservation planning. Genes. 2023;14(3):546. 36980819 10.3390/genes14030546 PMC10048327

[45] Johnson RN, O’Meally D, Chen Z, Etherington GJ, Ho SY, Nash WJ, et al. Adaptation and conservation insights from the koala genome. Nat Genet. 2018;50(8):1102–11. 29967444 10.1038/s41588-018-0153-5 PMC6197426

[46] Celniker SE, Rubin GM. The Drosophila melanogaster genome. Annu Rev Genomics Hum Genet. 2003;4(1):89–117. 14527298 10.1146/annurev.genom.4.070802.110323

[47] Rech GE, Radío S, Guirao-Rico S, Aguilera L, Horvath V, Green L, et al. Population-scale long-read sequencing uncovers transposable elements associated with gene expression variation and adaptive signatures in Drosophila. Nat Commun. 2022;13(1):1948. 35413957 10.1038/s41467-022-29518-8 PMC9005704

[48] Kapitonov VV, Jurka J. Molecular paleontology of transposable elements in the *Drosophila melanogaster* genome. Proc Natl Acad Sci U S A. 2003;100:6569–74. 10.1073/pnas.0732024100 PMC164487 12743378

[49] Stukenbrock EH, Jørgensen FG, Zala M, Hansen TT, McDonald BA, Schierup MH. Whole-genome and chromosome evolution associated with host adaptation and speciation of the wheat pathogen Mycosphaerella graminicola. PLoS Genet. 2010;6(12):e1001189. 21203495 10.1371/journal.pgen.1001189 PMC3009667

[50] Feurtey A, Lorrain C, McDonal MC, Milgate A, Solomon PS, Warren R, et al. A thousand-genome panel retraces the global spread and adaptation of a major fungal crop pathogen. Nat Commun. 2023;14:1059. 10.1038/s41467-023-36674-y PMC9958100 36828814

[51] Van Wyk S, Wingfield BD, De Vos L, Van der Merwe NA, Steenkamp ET. Genome-wide analyses of repeat-induced point mutations in the Ascomycota. Front Microbiol. 2021;11:622368. 33597932 10.3389/fmicb.2020.622368 PMC7882544

[52] Badet T, Oggenfuss U, Abraham L, McDonald BA, Croll D. A 19-isolate reference-quality global pangenome for the fungal wheat pathogen Zymoseptoria tritici. BMC Biol. 2020;18(12):1–18. 10.1186/s12915-020-0744-3 PMC7014611 32046716

[53] Weilguny L, Kofler R. DeviaTE: assembly-free analysis and visualization of mobile genetic element composition. Mol Ecol Resour. 2019;19(5):1346–54. 31056858 10.1111/1755-0998.13030 PMC6791034

[54] Li H, Durbin R. Fast and accurate long-read alignment with Burrows-Wheeler transform. Bioinformatics. 2010;26(5):589–95. 20080505 10.1093/bioinformatics/btp698 PMC2828108

[55] Li H, Handsaker B, Wysoker A, Fennell T, Ruan J, Homer N, et al. The Sequence Alignment/Map format and SAMtools. Bioinformatics. 2009;25(16):2078–9. 10.1093/bioinformatics/btp352 PMC2723002 19505943

[56] Quinlan AR, Hall IM. BEDTools: a flexible suite of utilities for comparing genomic features. Bioinforma (Oxford, England). 2010;26(6):841–2. 10.1093/bioinformatics/btq033 PMC2832824 20110278

[57] Chaisson MJ, Tesler G. Mapping single molecule sequencing reads using basic local alignment with successive refinement (BLASR): application and theory. BMC Bioinformatics. 2012;13(1):238. 22988817 10.1186/1471-2105-13-238 PMC3572422

[58] Edgar RC. MUSCLE: multiple sequence alignment with high accuracy and high throughput. Nucleic Acids Res. 2004;32(5):1792–7. 15034147 10.1093/nar/gkh340 PMC390337

[59] Wickham H. ggplot2: elegant graphics for data analysis. Basel: Springer Nature; 2016.

[60] Hoskins RA, Carlson JW, Wan KH, Park S, Mendez I, Galle SE, et al. The release 6 reference sequence of the Drosophila melanogaster genome. Genome Res. 2015;25(3):445–58. 25589440 10.1101/gr.185579.114 PMC4352887

[61] Kofler R. SimulaTE: simulating complex landscapes of transposable elements of populations. Bioinformatics. 2018;34(8):1419–20. 29186298 10.1093/bioinformatics/btx772 PMC5905622

[62] Camacho C, Coulouris G, Avagyan V, Ma N, Papadopoulos J, Bealer K, et al. BLAST+: architecture and applications. BMC Bioinformatics. 2009;10:1–9. 20003500 10.1186/1471-2105-10-421 PMC2803857

[63] Hall MB. Rasusa: randomly subsample sequencing reads to a specified coverage. J Open Source Softw. 2022;7(69):3941. 10.21105/joss.03941.

[64] GenomeDelta Pianezza R. Zenodo. 2024. 10.5281/zenodo.14215554.

